# Attribution retraining group therapy for outpatients with major depression disorder, generalized anxiety disorder, and obsessive-compulsive disorder: a pilot study^☆^

**DOI:** 10.1016/S1674-8301(11)60046-8

**Published:** 2011-09

**Authors:** Chun Wang, Jie Zhang, Jijun Li, Ning Zhang, Yalin Zhang

**Affiliations:** a Department of Medical Psychology, Nanjing Brain Hospital, Nanjing, Jiangsu 210029, China;; b Mental Health Institute, Second Xiangya Hospital, Central South University, Changsha,Wuhan 410011, China;; c Department of Psychological Counseling, Shandong University of Science and Technology, Qingdao, Shandong 266510, China.

**Keywords:** attribution retraining, group psychotherapy, major depression disorder, generalized anxiety disorder, obsessive-compulsive disorder

## Abstract

The aim of this present study is to examine the efficacy of attribution retraining group therapy (ARGT) and to compare the responses of outpatients with major depression disorder (MDD), generalized anxiety disorder (GAD) and obsessive-compulsive disorder (OCD). We carried out a prospective uncontrolled intervention study with a 8-weeks of ARGT on sixty three outpatients with MDD, GAD or OCD. Hamilton rating scale for depression, Hamilton rating scale for anxiety, Yale-Brown obsessive-compulsive scale, attribution style questionnaire, self-esteem scale, index of well-being, and social disability screening schedule were administered before and after treatment. Significant improvement in symptoms and psychological and social functions from pre- to posttreatment occurred for all participants. The changes favored MDD patients. Our study suggested that ARGT may improve the symptoms and psychological-social functions of MDD, GAD, and OCD patients. MDD patients showed the best response.

## INTRODUCTION

Attribution retraining (AR) is one of the therapeutic approaches within the larger group of cognitive behavior therapy. It is a therapy that treats clients' maladjusted emotions and behaviors by changing their explanations for problems and symptoms[1],[2]. AR has become popular because it is short-term, operable, and particularly reseach-supported in China in recent years[1],[3],[4]. People attribute behaviors and events to various reasons. Attributional style refers to a person's characteristic ways of explaining the causes of events[5], which is the basic concept of AR[4],[6]. There are three facets of how people can explain a situation: stable-unstable; global-local; and internal-external[7],[8]. In AR interventions, therapists usually target the patient's automatic thoughts, which are rooted in unhealthy attributions, and challenge these pessimistic attributions by means of offering alternative explanations premised upon healthy attributions[2].

AR is initially based on the integrated hopelessness/self-esteem theory proposed by Seligman, Abramson, and Metalsky[7]–[9]. They proposed a “depressive attributional style” comprised of internal, stable, and global attributions for negative events[7]. Many studies supported the idea that depressive attributions are at the core of most depressions[10]. Abramson, Metalsky and Alloy put forward a hopelessness theory that individuals with cognitive vulnerability (maladaptive attributional style), when experiencing negative life events, are likely to become hopeless and, in turn, develop depressive symptoms[8].

It has frequently been suggested that low self-esteem and low self-worth are closely related to psychological and emotional problems, particularly depression[11]–[14]. Metalsky, Joiner, Hardin and Abramson integrated the hopelessness and self-esteem theories of depression and proposed that the Attributional Diathesis×Low Self-Esteem×Failure interaction predicts enduring depressive reactions through the mediating role of hopelessness[9]. There is evidence of a vulnerability–stress component in which maladaptive attributional style and low self-esteem are hypothesized to interact with negative life events to contribute to the formation of hopelessness and depression[15]–[17].

Some studies indicated that maladaptive attributional styles predicted poor response to treatment for depression[18],[19]. It may be valuable to improve patients' attributional styles and self-esteem. Improvement in self-esteem has therefore regularly been described as one of the main treatment aims of ARGT. To date, almost all the published reports on AR based on integrated hopelessness/self-esteem theory are related to the use of AR with depressed patients[3],[20],[21]. However, according to the definition of AR, it can be applied to a variety of psychological problems with maladaptive attributional style[2]. There are two levels of indication of AR. Level 1: AR refers to people whose maladaptive attributional styles are the cause of their psychological disorders, such as depressed patients. Level 2: AR refers to people who hold maladaptive attributional styles. Changing maladaptive attributional styles is one of the approaches of psychotherapy[22]. Therefore AR could treat many mental disorders with maladaptive attributional styles in the two kinds of conditions.

Studies on anxiety and attributional style indicate that maladaptive attributional style is a nonspecific diathesis for symptoms of both anxiety and depression[23],[24] that predicts the onset of anxiety[25]. Alloy *et al*.[26] extend the hopelessness theory of depression and anxiety, relating the two disorders to the constructs of helplessness and negative outcome expectancy. Some published findings support the model[27],[28]. People in depression and anxiety are found to have low subjective[29],[30] and social function[31],[32]. Subjective well-being and social function are two important parts of life quality. There is increasing awareness that the goal of psychotherapy in mental disorders should not simply be a reduction in symptoms, but a restoration of psychological and social function and improvement in life quality. Those are all the aims of attribution retraining.

In China, Wang *et al*.[1],[33] developed a group form of attribution retraining named attribution retraining group therapy (ARGT) based on the integrated hopelessness/self-esteem theory and AR. ARGT is a form of group cognitive-behavior therapy that treats clients' emotions and behaviors by changing their maladaptive attributional styles and low self-esteem, and improves their life quality as well. They tested ARGT on depressed Chinese college graduates and outpatients with major depression disorder (MDD), generalized anxiety disorder (GAD) and obsessive-compulsive disorder (OCD) and demonstrated that ARGT can reduce their symptoms and change their maladaptive attributional styles[3],[4].

The primary purpose of the present study was to examine the effectiveness of ARGT for outpatients with MDD, GAD and OCD. The secondary purpose was to compare the response of ARGT in MDD, GAD and OCD. We hypothesized that: 1) ARGT will result in significant improvement in depression, anxiety, obsessive-compulsive symptoms, attribution style, self-esteem, well-being, and social function for treating MDD, GAD and OCD; 2) ARGT would yield the best outcomes for MDD outpatients in the three groups of patients because it was designed initially for depression.

## MATERIALS AND METHODS

### Participants

Sixty-three outpatients aged 16-50 years who met the DSM- IV criteria for MDD, GAD or OCD based upon the structured clinical interview for DSM-IV Axis I disorders, patient edition (SCID-I/P, Version 2.0)[34] were recruited from outpatients of a mental health hospital in Nanjing, China from 2007 to 2008. The diagnostic interview was implemented by two well trained clinicians experienced in SCID-I/P before patients entered the study. The other inclusion criteria were: the MDD diagnostic group: 1) the 24-item version of the Hamilton rating scale for depression (HAMD) score ≥18; 2) the GAD diagnostic group: the 14-item version of the Hamilton rating scale for anxiety (HAMA) score ≥14; 3) the OCD diagnostic group: Yale-Brown obsessive-compulsive scale (Y-BOCS) ≥ 16. The participant had to be willing to participate in ARGT for 8 sessions.

The exclusion criteria from the investigation were: 1) neurological disease; 2) serious physical illness (e.g. heart, lung, liver, kidney or blood system disease); 3) drug or alcohol abuse; 4) psychotic symptoms; 5)personality disorders; 6) pregnancy; 7) suicidal risk; 8) treatment by antidepressants or other psychotropic medications within 6 months prior to the start of the trial; 9) more than a single target diagnosis (e.g., comorbid for MDD and GAD or MDD and OCD).

Approval for this study was granted by the ethics committee of Nanjing Brain Hospital, Nanjing, China, prior to sample collection and informed written consent was received from all participants of this study.

### Measures

Demographic data (age, gender, marital status, education level, and family environment) and clinical characteristics (onset, stressful life events, course of disease, psychotropic medications history, psychotherapy history, family history, and physical illness history) were requested before commencing treatment.

The following scales were used to evaluate symptoms: The 24-item version of the HAMD[35] was used for measuring severity of depressive symptoms. Internal consistency of Chinese version is 0.88-0.99[36]. The 14-item version of the HAMA[37] was used to measure severity of anxiety symptoms. The inter-rater reliability of Chinese version is 0.93[36]. The 10-item Y-BOCS[38] was employed to assess severity of obsessions and compulsions only for OCD subjects. The inter-rater reliability is 0.75, test-retest reliability is 0.91[39]. The Attribution Style Questionnaire (ASQ)[40] was used to measure attributional style. It is divided into 3 subscales: internal-external (internality), global-specific (globality), and stable-unstable (stability) dimensions. Lower scores indicate a higher degree of maladaptation in attributional style. The hopelessness score of the ASQ is calculated by adding the globality-dimension scores of negative events to the stability-dimension scores of negative events and then dividing by 2. Lower scores show milder hopelessness. Cronbach alpha reliability of the Chinese version is 0.84, with 0.49, 0.79, and 0.82 for the three subscales[41]. The Self-Esteem Scale (SES)[42] was used to evaluate self-esteem. This self-rating scale consists of 10 items with responses to items ranging from 1 (strongly agree) to 4 (strongly disagree). Higher total scores indicate lower self-esteem. The split-half reliability of Chinese version is 0.96. Inter-rater reliability is 0.78. The correlation coefficient with confidence is 0.65[36]. The index of well-being (IWB)[43] was used to evaluate psychological well-being. IWB is a nine-item semantic differential scale assessing the cognitive (1 item) and affective dimensions (8 items) of psychological well-being. Higher total scores indicate higher psychological well-being. The Cronbach alpha reliability of the affective dimensions is 0.89, and test-retest reliability is 0.43. The correlation coefficient between the two dimensions is 0.65[36]. The 10-item social disability screening schedule (SDSS)[44] was used for measuring social function. It is a shortened version of psychiatric disability assessment schedule (DAS). Higher total scores indicate lower social function. The inter-rater reliability is 0.85-0.99[44].

Measurement points were at week 0 and 8 (posttest). Symptoms measurements (HAMD, HAMA and Y-BOCS) were designed as primary outcomes; psychological and social functions (ASQ, SES, IWB, and SDSS) measurements were secondary outcomes. HAMA, HAMD, and Y-BOCS were administered to each patient by two psychologists independently who did not know the purpose of the study. Before administering any assessments, the two psychologists received professional training specific to the assessments. The Spearman correlation coefficients (HAMA, HAMD, and Y-BOCS) between the two psychologists were 0.84, 0.83, and 0.93, respectively.

### Procedure and treatment

ARGT in the present study consisted of 8 two-h sessions, carried out once a week according to a treatment manual for ARGT[33], which was previously tested[3]. Participants were divided into 8 ARGT groups according to the sequence of entering the study, not their diagnosis. Within a structured therapy program, each session had a relatively stable subject: ① knowing and supporting each other; ② the meaning of symptoms and the effects of cognitive factors; ③ the role of attribution in psychology; ④ participants' upbringing and basic beliefs; ⑤ rebuilding attributional styles and practicing new behaviors; ⑥ consolidating new attribution styles and behaviors; ⑦ self-esteem, personality and attributions for positive events; ⑧ sharing future plans and discussing leaving.

All sessions were conducted by two therapists: one main therapist who was for all groups, assisted by a co-therapist who varied according to the ARGT group. All therapists had completed training in psychotherapy. The main therapist was a registered clinical and counseling psychologist in Professional Organizations and Individual Practitioners in Clinical and Counseling Psychology, Chinese Psychological Society. The co-therapists were kept blind with respect to the study purpose. To control treatment integrity, the co-therapist recorded whether each step of the intervention plan occurred or did not occur and discussed with the main therapist before the next session. In addition, the interventions were supervised by 4 registered psychologist supervisors from Chinese Psychological Society. One ARGT group was supervised by one psychologist supervisor once every two weeks.

### Statistical analysis

Data analyses were performed using SPSS 15.0 for Windows statistical software (SPSS Inc., Chicago, IL, USA). Baseline data were compared between diagnostic groups in relation to demographic variables, independent-samples *t* test for continuous variables and the χ^2^ test for nominal variables. A multivariate analysis of covariance (MANCOVA) model was used to to analyze the repeated data of symtoms (HAMD, HAMA, Y-BOCS) and psychological-social functions (ASQ, SES, IWB, SDSS). Reported multivariate *F* values are based on Pillai's Trace. If multivariate effects of condition were significant, we conducted univariate comparisons. Reductions in scores were compared between the three groups using univariate analysis of covariance (ANCOVA). The paired-samples *t* test was used to compare the scores of continuous variables at baseline and week 8 in each group.

The effect size was calculated by the Cohen formula [*d* = (*M*_1_ - *M*_2_) / SD][45]. In multivariate and univariate analysis, the partial eta-squared [*ηp*^2^ = *SS*_effect_ / (*SS*_effect_ + *SS*_error_)] was used as measure of effect size. The following rules of thumb for *ηp*^2^ have emerged: small = 0.01; medium = 0.06; large = 0.14[46].

## RESULTS

### Baseline data

The demographic and clinical characteristics of 54 patients at baseline are given in ***Table 1***. The three groups were well-matched in age, gender, marital status, family environment, onset, course of disease and family history. They differed in educational level, stressful life events, history of psychotropic medications, and history of physical illness. So educational level, stressful life events, history of psychotropic medications and history of physical illness were regarded as covariates in the analysis of variance. At week 0, 63 eligible outpatients attended the study, including those in the MDD group (*n* = 21), the GAD group (*n* = 23), and the OCD group (*n* = 19). At week 8, 9 outpatients dropped out (14.3%), with 2 of the outpatients due to time and space limitation, 2 for not accepting psychotherapy, 2 for needing other treatments because of suicide risk, 2 for continuous absence for two sessions, and 1 for wrong diagnosis. At the end of ARGT, 90.5% of the MDD group, 82.6% of the GAD group and 84.2% the OCD group completed the study. The difference was not statistically significant (χ^2^ = 0.64, *P* = 0.73).

### Treatment outcomes in symptoms and psychological-social functions

By a MANOVA, significant improvement in symptoms (HAMD, HAMA, and Y-BOCS) and psychological-social functions (ASQ, SES, IWB, and SDSS) from pre- to posttreatment occurred for all participants and univariate comparisons for the three scales were significant (***Table 2***). ***Table 3*** presents the paired-samples *t* test outcomes of pre-post scores and effect sizes of psychological and social rating scales. After treatment, the GAD group did not improve significantly in hopelessness and subjective well-being. There were no significant statistical differences in subjective well-being in the OCD group. Significant changes were found for each of the remaining measures. According to Cohen, the effects were medium to large-sized in the MDD group (*d* > 0.5). Changes were small-effect in hopelessness, subjective well-being in the GAD group, and in subjective well-being in the OCD group.

### Comparability of score reductions between MDD, GAD and OCD groups

When the reductions in scores were compared between the three diagnosis groups, using educational level, stressful life events, psychotropic medications history and physical illness history (the three groups were not matched) and the baseline scores as covariates, significant differences were found in the reduction of HAMD scores (*F* = 2.59, *P* = 0.09, *ηp*^2^ = 0.10, pairwise comparison showed that the estimates marginal mean of MDD group was more than OCD group). There were no significant differences in reductions among three groups in HAMA, ASQ, hopelessness, IWB, SES, and SDSS (*P* > 0.05).

## DISCUSSION

In the present study, all the three groups of patients improved significantly in depression and anxiety, and obsessive-compulsive symptoms were reduced significantly in OCD patients after 8 weeks of ARGT. This suggests that ARGT exerts a positive effect on MDD, GAD and OCD patients, which agrees with the findings from previous studies[3],[20],[21]. At the same time, effective promotion of social function was found in all three groups of patients, suggesting that ARGT might assist them in readjusting to society. In this study, patients diagnosed with MDD, GAD and OCD exhibited positive change in attributional style after ARGT. As a basic concept of therapy, ARGT seems to change maladaptive attributional styles to more adaptive ones. Self-esteem has been discussed as an approach to relieve symptoms and improve psychological function[33]. The results of this study suggest that ARGT seems to be effective in improving self-esteem for MDD, GAD and OCD patients. Low self-esteem is closely related to psychiatric disorders. It frequently accompanies depression and anxiety[13],[47]. Additionally, a positive association between self-esteem and well-being is found, and high self-esteem has also been reported to be one of the strongest predictors of well-being[48].

**Table 1:** Demographic and clinical characteristics of the study participants

	MDD (*n*=19)	GAD (*n*=19)	OCD (*n*=16)	Statistics	*P*
Age (y)				1.534^a^	0.225
	27.26±7.48	33.11±10.09	27.25±10.98	2.311^b^	0.109
Sex [*n*(%)]					
Male	8 (42.1)	9(47.4)	7(43.8)	0.111^c^	0.946
Female	11(57.9)	10(52.6)	9(56.3)		
Marital status [*n*(%)]					
Married	7(36.8)	12(63.2)	4(25.0)	7.187^d^	0.070
Never married	11(57.9)	6(31.6)	12(75.0)		
Divorced	1(5.3)	1(5.3)	0(0)		
Widowed	0(0)	0(0)	0(0)		
Educational level [*n*(%)]					
< 9 y	1(5.3)	0(0)	2(12.5)	12.753^d^	0.020
9-12 y	2(10.5)	8(42.1)	2(12.5)		
12-16 y	14(73.7)	7(36.8)	12(75.0)		
> 16 y	2(10.5)	4(21.1)	0(0)		
Family environment [*n*(%)]					
City	15(78.9)	14(73.7)	11(68.8)	2.394^d^	0.847
Countryside	4(21.1)	4(26.3)	4(25.0)		
Others	0(0)	0(0)	1(6.3)		
Onset [*n*(%)]					
First	14(73.7)	13(68.4)	11(68.8)	0.244^d^	1.000
Recurrence	5(26.3)	6(31.6)	5(31.3)		
Stressful life events [*n*(%)]					
Yes	17(89.5)	18(94.7)	9(56.3)	8.369^d^	0.014
No	2(10.5)	1(5.3)	7(43.8)		
Course of disease [*n*(%)]					
≤1 y	7(36.8)	1(5.3)	5(31.3)	8.889^d^	0.051
1-10 y	11(57.9)	15(78.9)	7(43.8)		
≥10 y	1(5.3)	3(15.8)	4(25.0)		
Psychotropic medications history [*n*(%)]					
Yes	4(21.1)	10(52.6)	10(52.6)	6.839^c^	0.033
No	15(78.9)	9(47.4)	6(47.4)		
Psychotherapy history [*n*(%)]					
Yes	4(21.1)	4(21.1)	4(25.0)	0.221^d^	1.000
No	15(78.9)	15(78.9)	12(75.0)		
Family history [*n*(%)]					
Yes	4(21.1)	3(15.8)	6(37.5)	2.264^d^	0.378
No	15(78.9)	16(84.2)	10(62.5)		
Physical illness history [*n*(%)]					
Yes	6(31.6)	9(47.4)	1(6.3)	7.318^d^	0.020
No	13(68.4)	10(52.6)	15(93.8)		

a: Levene test; b: *F* test; c: Pearson Chi-Square test; d: Fisher's exact test. MDD: major depression disorder; GAD: generalized anxiety disorder; OCD: obsessive-compulsive disorder.

**Table 2:** MANOVA and univariate comparisons outcomes on symtoms and psychological-social functions between baseline and after treatment

measures	MDD (*n*=19)		GAD (*n*=19)		OCD (*n*=16)		Statistical results			
	pre	post	pre	post	pre	post	F(pre**-**post)	*P*	*ηp*^2^	observed power
Symtoms							45.03	<0.05	0.91	1.00
HAMD	27.95 ± 9.62	2.79 ± 2.68	21.58 ± 5.97	3.42 ± 1.87	20.25 ± 6.87	4.06 ± 3.21	95.38	<0.05	0.87	1.00
HAMA	21.42 ± 5.96	2.47 ± 2.41	22.58 ± 6.09	3.89 ± 2.45	15.25 ± 7.22	2.31 ± 1.96	135.56	<0.05	0.90	1.00
Y-BOCS	-	-	-	-	26.06 ± 6.42	15.94 ± 4.73	29.87	<0.05	0.67	1.00
Psychological and social functions							47.37	<0.05	0.83	1.00
ASQ	0.08 ± 1.24	1.01 ± 0.88	0.46±0.85	1.36 ± 0.84	0.54 ± 0.85	1.46 ± 0.91	7.93	0.007	0.13	0.79
Hopelessness	4.73 ± 1.22	3.86 ± 1.22	3.93 ± 0.89	3.71 ± 1.18	4.40 ± 0.90	3.45 ± 0.84	14.36	<0.05	0.22	0.96
IWB	6.83 ± 1.66	9.20 ± 1.36	8.88±1.65	9.22 ± 1.54	8.25 ± 2.27	9.27 ± 1.28	21.92	<0.05	0.30	1.00
SES	26.26 ± 4.41	21.47 ± 3.50	24.53 ± 3.95	21.00 ± 4.23	24.63 ± 3.34	21.81 ± 3.92	47.88	<0.05	0.48	1.00
SDSS	7.26 ± 2.02	0.37 ± 0.68	6.37 ± 4.36	1.00 ± 0.94	6.13 ± 3.16	1.06 ± 1.53	175.10	<0.05	0.77	1.00

MDD: major depression disorder; GAD: generalized anxiety disorder; OCD: obsessive-compulsive disorder; HAMD: Hamilton rating scale for depression; HAMA: Hamilton rating scale for anxiety; Y-BOCS: Yale-Brown obsessive-compulsive Scale; ASQ: attribution style questionnaire; SES: self-esteem scale; IWB: index of well-being; SDSS: social disability screening schedule. (Mean±SD)

**Table 3:** Comparison of mean scores on psychological and social rating scales at baseline and after treatment

	MDD			GAD			OCD		
	*t*	*P*	*d*	*t*	*P*	*d*	*t*	*P*	*d*
ASQ	-3.688	0.002	-0.750	-4.162	0.001	-1.059	-4.386	0.001	-1.082
Hopelessness	2.897	0.010	0.713	0.789	0.440	0.247	2.679	0.017	1.056
SES	7.619	0.000	1.086	2.950	0.009	0.894	3.419	0.004	0.844
IWB	-5.040	0.000	-1.428	-0.931	0.364	-0.206	-1.880	0.080	-0.449
SDSS	-3.823^a^	0.000	3.411	5.806	0.000	1.232	6.702	0.000	1.604

MDD: major depression disorder; GAD: generalized anxiety disorder; OCD: obsessive-compulsive disorder; ASQ: attribution style questionnaire; SES: self-esteem scale; IWB: index of well-being; SDSS: social disability screening schedule.

With regard to emotions, the MDD group showed significant improvement in subjective well-being and hopelessness; OCD patients experienced a significant reduction in hopelessness but no significant increase in subjective well-being; GAD patients improved on neither. When baseline scores and covariates were partialled out, the changes from baseline were similar in MDD, GAD and OCD. These data suggest that improvement of positive emotions did not occur at the same time as reduction of negative emotions in ARGT. It is understood that hopelessness is usually better correlated with depression than anxiety[49]. In addition, well-being improved significantly only in MDD patients, implying that ARGT is statistically significantly favoring MDD. Ryff *et al*'s study[50]. suggest that the absence of well-being creates conditions of vulnerability to possible future adversities and that the route of recovery lies not exclusively in alleviating the negative, but in engendering the positive.

Based on the above discussion, ARGT yields the best outcomes for MDD patients in the three groups, which is in line with the second hypothesis. One explanation for the finding is that different diagnostic groups demonstrated different dose-effect patterns[51]. Howard *et al*.[52] found that depressed patients responded most quickly to psychotherapy, followed by anxious patients, while borderline-psychotic patients responded at the slowest rate. Another explanation is that ARGT originates from the integrated hopelessness/self-esteem theory for depression and was designed initially for depression. So it is more suitable for MDD than other mental disorders. It is proposed that other psychopathological factors may be integrated into ARGT to improve mental health more effectively for patients with various mental disorders.

There are shortcomings in this study. First, it is limited because it reflects the effectiveness of ARGT for MDD, GAD and OCD in an uncontrolled trial. Fortunately, our prior studies have proven that the improvements were due to treatment factors, not natural self-improvement over time[3],[4]. Some other studies also suggested that the waiting group yielded a small to medium effect size and treatment group gained a large effect size[53],[54]. Second, this study only evaluated changes over a short time period, making the long term effectiveness of ARGT for patients. Furthermore, a small sample size and diagnositic heterogeneity are also limitations of this study.

In summary, ARGT may improve symptoms and psychological-social functions for MDD, GAD, and OCD. The effectiveness of ARGT may include the following aspects: 1) reduction of symptoms and recovery of social function; 2) improvement in attributional styles and self-esteem; 3) elimination of hopelessness and enhancement of experience of well-being. With an all-round improvement in clinical symptoms, psychological function, and social function, ARGT can help patients not only to reduce symptomatic distress and readjust to society, but also to positively restructure their psychological function. This could aid in facing life after treatment and in reducing relapses. MDD patients respond best to ARGT, followed by GAD and OCD patients. In future studies, a control group assigned to a different treatment condition (preferably through randomization) and longer follow-up intervals should be used to propose further clinical indications of ARGT.
